# ID 220 - Análise de Impacto Orçamentário da Incorporação do Sistema Flash de Monitorização de Glicemia na Perspectiva do Sistema Único de Saúde (SUS)

**DOI:** 10.5327/2237-9622.2025.v34s1.220

**Published:** 2025-11-25

**Authors:** Mariana Andrades Fiorini Monteiro Novo, Vania dos Santos Nunes Nogueira, Julia Simões Correa Galendi

**Keywords:** análise de impacto orçamentário, diabetes mellitus, sistema flash de monitoramento de glicemia

## Abstract

**Introdução:**

O diabetes mellitus (DM) é uma doença metabólica caracterizada por hiperglicemias persistentes resultantes de defeitos na secreção ou na ação da insulina, ou envolvendo ambos os mecanismos. O sistema flash de monitorização da glicose por escaneamento intermitente (SFMG) é uma abordagem menos invasiva e mais conveniente de monitorização contínua da glicose em indivíduos com DM. O objetivo desse estudo foi estimar as consequências econômicas da incorporação do SFMG para a monitorização de pacientes com DM1 com hipoglicemia grave ou noturna ou DM2 que usam múltiplas doses de insulina e com histórico de hipoglicemia grave na perspectiva do SUS.

**Método:**

Foi realizada uma análise de impacto orçamentário (AIO), a perspectiva foi a do SUS, a população-alvo foi pacientes com DM1 com hipoglicemia grave ou noturna ou DM2 em uso de insulina e com histórico de hipoglicemia grave, e foram considerados dois cenários: o uso do SFMG, e o comparador foi a utilização do automonitoramento de glicemia capilar (AMGC). Foi elaborado um modelo estático no Microsoft Office Excel (Microsoft Corporation, Redmond, WA, EUA), seguindo as Diretrizes Metodológicas de Análise de Impacto Orçamentário do Ministério da Saúde. O horizonte temporal foi de cinco anos. Foram considerados custos relacionados ao uso do SFMG e do AMGC, custos diretos do tratamento hospitalar de eventos agudos de hipoglicemia e cetoacidose. O considerou dois cenários de penetração, ambos com difusão inicial de 20% no primeiro ano. O cenário de penetração conservador considerou incrementos de 5% por ano, e o cenário de penetração acelerado considerou 10% por ano. Ao final do quinto ano, o cenário de penetração conservador alcançou 40%, e o acelerado 60% do mercado.

**Resultados:**

Foram calculados o impacto orçamentário para quatro cenários de subgrupos de pacientes com hipoglicemia grave ou hipoglicemia noturna exclusiva para os pacientes com DM1 e hipoglicemia grave para os pacientes com DM2, que são subgrupos de pacientes que, em teoria, beneficiariam-se mais da tecnologia. Para a população de pacientes com DM1, o impacto orçamentário calculado ficou entre R$ 5.952.151.059,00 e R$ 7.932.343.524,00, e para os pacientes com DM2 em uso de múltiplas doses de insulina foi entre R$ 7.906.417.177,00 e R$ 10.536.834.540,00, considerando uma taxa de penetração conservadora ou acelerada respectivamente. Nos cenários avaliando subgrupos, o impacto orçamentário para o conjunto de pacientes DM2 e DM1 com hipoglicemia severa variou entre R$ 2.362.934.843,00 e R$ 3.149.057.569,00, considerando a penetração conservadora ou acelerada. Nos cenários avaliando o conjunto de pacientes DM2 com hipoglicemia severa e DM1 com hipoglicemia noturna exclusiva, o impacto orçamentário variou de R$ 2.060.927.949,93 a R$ 2.932.887.912,00, considerando a penetração conservadora ou acelerada.

**Conclusão:**

Foram acrescentados nesta avaliação todos os indivíduos com DM1 e DM2 em insulinoterapia, e a determinação da população elegível foi o que mais influenciou no impacto orçamentário.

